# A comprehensive study of common and rare genetic variants in spermatogenesis-related loci identifies new risk factors for idiopathic severe spermatogenic failure

**DOI:** 10.1093/hropen/hoae069

**Published:** 2024-11-13

**Authors:** Andrea Guzmán-Jiménez, Sara González-Muñoz, Miriam Cerván-Martín, Nicolás Garrido, José A Castilla, M Carmen Gonzalvo, Ana Clavero, Marta Molina, Saturnino Luján, Samuel Santos-Ribeiro, Miguel Ángel Vilches, Andrea Espuch, Vicente Maldonado, Noelia Galiano-Gutiérrez, Esther Santamaría-López, Cristina González-Ravina, Fernando Quintana-Ferraz, Susana Gómez, David Amorós, Luis Martínez-Granados, Yanira Ortega-González, Miguel Burgos, Iris Pereira-Caetano, Ozgur Bulbul, Stefano Castellano, Massimo Romano, Elena Albani, Lluís Bassas, Susana Seixas, João Gonçalves, Alexandra M Lopes, Sara Larriba, Rogelio J Palomino-Morales, F David Carmona, Lara Bossini-Castillo

**Affiliations:** Departamento de Genética e Instituto de Biotecnología, Centro de Investigación Biomédica (CIBM), Universidad de Granada, Granada, Spain; Instituto de Investigación Biosanitaria ibs. GRANADA, Granada, Spain; Departamento de Genética e Instituto de Biotecnología, Centro de Investigación Biomédica (CIBM), Universidad de Granada, Granada, Spain; Instituto de Investigación Biosanitaria ibs. GRANADA, Granada, Spain; Institute of Parasitology and Biomedicine López-Neyra (IPBLN), CSIC, Granada, Spain; IVIRMA Global Research Alliance, IVI Foundation, Instituto de Investigación Sanitaria La Fe (IIS La Fe), Valencia, Spain; Instituto de Investigación Biosanitaria ibs. GRANADA, Granada, Spain; Departamento de Anatomía y Embriología Humana, Facultad de Medicina, Universidad de Granada, Granada, Spain; Instituto de Investigación Biosanitaria ibs. GRANADA, Granada, Spain; Unidad de Reproducción, UGC Obstetricia y Ginecología, HU Virgen de las Nieves, Granada, Spain; Instituto de Investigación Biosanitaria ibs. GRANADA, Granada, Spain; Unidad de Reproducción, UGC Obstetricia y Ginecología, HU Virgen de las Nieves, Granada, Spain; Instituto de Investigación Biosanitaria ibs. GRANADA, Granada, Spain; Unidad de Reproducción, UGC Obstetricia y Ginecología, HU Virgen de las Nieves, Granada, Spain; Servicio de Urología, Hospital Universitari i Politecnic La Fe e Instituto de Investigación Sanitaria La Fe (IIS La Fe), Valencia, Spain; IVI-RMA Lisbon, Lisbon, Portugal; Department of Obstetrics and Gynecology, Faculty of Medicine, University of Lisbon, Lisbon, Portugal; Ovoclinic & Ovobank, Clínicas de Reproducción Asistida y Banco de óvulos, Marbella, Málaga, Spain; Hospital Universitario Torrecárdenas, Unidad de Reproducción Humana Asistida, Almería, Spain; UGC de Obstetricia y Ginecología, Complejo Hospitalario de Jaén, Jaén, Spain; VIDA RECOLETAS Seville, Seville, Spain; VIDA RECOLETAS Seville, Seville, Spain; IVIRMA Global Research Alliance, IVI Foundation, Instituto de Investigación Sanitaria La Fe (IIS La Fe), Valencia, Spain; IVIRMA Global Research Alliance, IVI Foundation, Instituto de Investigación Sanitaria La Fe (IIS La Fe), Valencia, Spain; IVIRMA Global Research Alliance, IVI Foundation, Instituto de Investigación Sanitaria La Fe (IIS La Fe), Valencia, Spain; IVIRMA Global Research Alliance, IVI Foundation, Instituto de Investigación Sanitaria La Fe (IIS La Fe), Valencia, Spain; Hospital Universitario Príncipe de Asturias, Alcalá de Henares, Madrid, Spain; Unidad de Urología, Hospital Universitario de Canarias, Santa Cruz de Tenerife, Spain; Departamento de Genética e Instituto de Biotecnología, Centro de Investigación Biomédica (CIBM), Universidad de Granada, Granada, Spain; Departamento de Genética Humana, Instituto Nacional de Saúde Dr Ricardo Jorge, Lisbon, Portugal; Division of Gynecology and Reproductive Medicine, Department of Gynecology, Fertility Center, Humanitas Research Hospital, IRCCS, Milan, Italy; Division of Gynecology and Reproductive Medicine, Department of Gynecology, Fertility Center, Humanitas Research Hospital, IRCCS, Milan, Italy; Division of Gynecology and Reproductive Medicine, Department of Gynecology, Fertility Center, Humanitas Research Hospital, IRCCS, Milan, Italy; Department of Biomedical Sciences, Humanitas University, Milan, Italy; Division of Gynecology and Reproductive Medicine, Department of Gynecology, Fertility Center, Humanitas Research Hospital, IRCCS, Milan, Italy; Laboratory of Seminology and Embryology, Andrology Service-Fundació Puigvert, Barcelona, Spain; i3S—Instituto de Investigação e Inovação em Saúde, Universidade do Porto, Porto, Portugal; Institute of Molecular Pathology and Immunology of the University of Porto (IPATIMUP), Porto, Portugal; Departamento de Genética Humana, Instituto Nacional de Saúde Dr Ricardo Jorge, Lisbon, Portugal; ToxOmics—Centro de Toxicogenómica e Saúde Humana, Nova Medical School, Lisbon, Portugal; Institute of Molecular Pathology and Immunology of the University of Porto (IPATIMUP), Porto, Portugal; CGPP-IBMC—Centro de Genética Preditiva e Preventiva, Instituto de Biologia Molecular e Celular, Universidade do Porto, Porto, Portugal; Human Molecular Genetics Group, Bellvitge Biomedical Research Institute (IDIBELL), L’Hospitalet de Llobregat, Barcelona, Spain; Instituto de Investigación Biosanitaria ibs. GRANADA, Granada, Spain; Departamento de Bioquímica y Biología Molecular I, Universidad de Granada, Granada, Spain; Departamento de Genética e Instituto de Biotecnología, Centro de Investigación Biomédica (CIBM), Universidad de Granada, Granada, Spain; Instituto de Investigación Biosanitaria ibs. GRANADA, Granada, Spain; Departamento de Genética e Instituto de Biotecnología, Centro de Investigación Biomédica (CIBM), Universidad de Granada, Granada, Spain; Instituto de Investigación Biosanitaria ibs. GRANADA, Granada, Spain

**Keywords:** spermatogenesis, idiopathic spermatogenic failure, genetics, male infertility, polygenic susceptibility, monogenic mutations

## Abstract

**STUDY QUESTION:**

Can genome-wide genotyping data be analysed using a hypothesis-driven approach to enhance the understanding of the genetic basis of severe spermatogenic failure (SPGF) in male infertility?

**SUMMARY ANSWER:**

Our findings revealed a significant association between SPGF and the *SHOC1* gene and identified three novel genes (*PCSK4*, *AP3B1*, and *DLK1*) along with 32 potentially pathogenic rare variants in 30 genes that contribute to this condition.

**WHAT IS KNOWN ALREADY:**

SPGF is a major cause of male infertility, often with an unknown aetiology. SPGF can be due to either multifactorial causes, including both common genetic variants in multiple genes and environmental factors, or highly damaging rare variants. Next-generation sequencing methods are useful for identifying rare mutations that explain monogenic forms of SPGF. Genome-wide association studies (GWASs) have become essential approaches for deciphering the intricate genetic landscape of complex diseases, offering a cost-effective and rapid means to genotype millions of genetic variants. Novel methods have demonstrated that GWAS datasets can be used to infer rare coding variants that are causal for male infertility phenotypes. However, this approach has not been previously applied to characterize the genetic component of a whole case–control cohort.

**STUDY DESIGN, SIZE, DURATION:**

We employed a hypothesis-driven approach focusing on all genetic variation identified, using a GWAS platform and subsequent genotype imputation, encompassing over 20 million polymorphisms and a total of 1571 SPGF patients and 2431 controls. Both common (minor allele frequency, MAF > 0.01) and rare (MAF < 0.01) variants were investigated within a total of 1797 loci with a reported role in spermatogenesis. This gene panel was meticulously assembled through comprehensive searches in the literature and various databases focused on male infertility genetics.

**PARTICIPANTS/MATERIALS, SETTING, METHODS:**

This study involved a European cohort using previously and newly generated data. Our analysis consisted of three independent methods: (i) variant-wise association analyses using logistic regression models, (ii) gene-wise association analyses using combined multivariate and collapsing burden tests, and (iii) identification and characterisation of highly damaging rare coding variants showing homozygosity only in SPGF patients.

**MAIN RESULTS AND THE ROLE OF CHANCE:**

The variant-wise analyses revealed an association between SPGF and *SHOC1*-rs12347237 (*P *=* *4.15E−06, odds ratio = 2.66), which was likely explained by an altered binding affinity of key transcription factors in regulatory regions and the disruptive effect of coding variants within the gene. Three additional genes (*PCSK4*, *AP3B1*, and *DLK1*) were identified as novel relevant players in human male infertility using the gene-wise burden test approach (*P *<* *5.56E−04). Furthermore, we linked a total of 32 potentially pathogenic and recessive coding variants of the selected genes to 35 different cases.

**LARGE SCALE DATA:**

Publicly available via GWAS catalog (accession number: GCST90239721).

**LIMITATIONS, REASONS FOR CAUTION:**

The analysis of low-frequency variants presents challenges in achieving sufficient statistical power to detect genetic associations. Consequently, independent studies with larger sample sizes are essential to replicate our results. Additionally, the specific roles of the identified variants in the pathogenic mechanisms of SPGF should be assessed through functional experiments.

**WIDER IMPLICATIONS OF THE FINDINGS:**

Our findings highlight the benefit of using GWAS genotyping to screen for both common and rare variants potentially implicated in idiopathic cases of SPGF, whether due to complex or monogenic causes. The discovery of novel genetic risk factors for SPGF and the elucidation of the underlying genetic causes provide new perspectives for personalized medicine and reproductive counselling.

**STUDY FUNDING/COMPETING INTEREST(S):**

This work was supported by the Spanish Ministry of Science and Innovation through the Spanish National Plan for Scientific and Technical Research and Innovation (PID2020-120157RB-I00) and the Andalusian Government through the research projects of ‘Plan Andaluz de Investigación, Desarrollo e Innovación (PAIDI 2020)’ (ref. PY20_00212) and ‘Proyectos de Investigación aplicada FEDER-UGR 2023’ (ref. C-CTS-273-UGR23). S.G.-M. was funded by the previously mentioned projects (ref. PY20_00212 and PID2020-120157RB-I00). A.G.-J. was funded by MCIN/AEI/10.13039/501100011033 and FSE ‘El FSE invierte en tu futuro’ (grant ref. FPU20/02926). IPATIMUP integrates the i3S Research Unit, which is partially supported by the Portuguese Foundation for Science and Technology (FCT), financed by the European Social Funds (COMPETE-FEDER) and National Funds (projects PEstC/SAU/LA0003/2013 and POCI-01-0145-FEDER-007274). S.S. is supported by FCT funds (10.54499/DL57/2016/CP1363/CT0019), ToxOmics-Centre for Toxicogenomics and Human Health, Genetics, Oncology and Human Toxicology, and is also partially supported by the Portuguese Foundation for Science and Technology (UIDP/00009/2020 and UIDB/00009/2020). S. Larriba received support from Instituto de Salud Carlos III (grant: DTS18/00101), co-funded by FEDER funds/European Regional Development Fund (ERDF)—a way to build Europe) and from ‘Generalitat de Catalunya’ (grant 2021SGR052). S. Larriba is also sponsored by the ‘Researchers Consolidation Program’ from the SNS-Dpt. Salut Generalitat de Catalunya (Exp. CES09/020). All authors declare no conflict of interest related to this study.

WHAT DOES THIS MEAN FOR PATIENTS? Our research aims to investigate the genetic causes of male infertility leading to very low sperm counts or a total absence of sperm in semen. We examined a large number of genetic variations in specific genes related to sperm production and we identified several genetic markers that are more common in affected men. Our findings highlight the key role of genetics in male infertility problems and provide cost-effective means for future research and diagnosis. This work brings us closer to understanding male infertility and will help to develop tools for personalized medicine.

## Introduction

Human reproductive health is a major concern worldwide, as infertility impacts one in seven couples (Sang *et al.*, 2023) and approximately one in every six people worldwide (WHO, 2023), with male factors contributing into around 50% of the cases (Eisenberg *et al.*, 2023). While obstructive causes of male infertility have been identified in some instances, a large proportion of them are attributed to spermatogenesis anomalies (Schlegel *et al.*, 2021). In this context, severe spermatogenic failure (SPGF) represents the most extreme non-obstructive manifestation of male infertility, implying a significant challenge for Assisted Reproductive Technology (ART) (Leaver, 2016). It is widely known that genetic factors are directly involved in the development of ∼25–30% of SPGF cases, including karyotype alterations, Y chromosome microdeletions, and high-penetrance point mutations in genes related to spermatogenesis (Chen *et al.*, 2020; Kasak *et al.*, 2022; Nagirnaja *et al.*, 2022; Tang *et al.*, 2022; Khan *et al.*, 2023; Lillepea *et al.*, 2024; Sieper *et al.*, 2024).

It should be considered that the intricate nature of spermatogenesis, which involves the combined and highly controlled expression of a wide spectrum of molecular pathways (Krausz *et al.*, 2015), provides a natural source of heterogeneity. As a consequence, a variety of genetic alterations can lead to SPGF (Stallmeyer *et al.*, 2024). Moreover, it is known that epigenetic factors, such as altered DNA methylation and histone modifications in key loci or deregulated miRNA expression, are strongly associated with male infertility (Rotondo *et al.*, 2021; Wagner *et al.*, 2023). Nevertheless, the current genetic diagnosis strategies for this manifestation of male infertility rely only on karyotype and Y chromosome deletion analyses, which are sometimes combined with whole exome or whole genome sequencing (WES/WGS) to identify rare mutations that cause monogenic forms of SPGF (Houston *et al.*, 2021). Unfortunately, WES and WGS technologies are expensive processes, and their results are sometimes hard to interpret (Krausz *et al.*, 2018; Tuttelmann *et al.*, 2018; Guerri *et al.*, 2019). Therefore, a genetic determinant is not established in most cases of SPGF diagnosis, and the aetiology of these infertile men is defined as unknown or idiopathic (Cannarella *et al.*, 2019).

Interestingly, cumulative knowledge clearly suggests that a significant proportion of this idiopathic form of SPGF may represent a multifactorial trait, influenced by common genetic variants of the human genome in combination with environmental factors (Cervan-Martin *et al.*, 2024). In this regard, the genome-wide association study (GWAS) strategy is a valuable tool for investigating the genetic component of complex phenotypes. GWASs are hypothesis-free approaches based on microarrays that allow the genotyping of millions of genetic variants, particularly single-nucleotide polymorphisms (SNPs). Recently, the latest GWAS performed on SPGF in a large European cohort revealed immune and spermatogenesis-related loci involved in the development of extreme patterns of male infertility (Cervan-Martin *et al.*, 2022). Furthermore, taking advantage of the current vast imputation reference panels that allow inference of very rare known haplotypes, the use of GWAS genotype information has been proposed as a feasible alternative to massively parallel sequencing approaches to study the monogenic causes of male infertility (Taliun *et al.*, 2021). In fact, the analysis of this type of dataset successfully led to the identification of damaging rare variants present in compound heterozygosis that caused male infertility in a non-consanguineous family affected by globozoospermia (Lopez-Rodrigo *et al.*, 2022).

Taking the above into consideration, we leveraged the previously mentioned European SPGF GWAS dataset (Cervan-Martin *et al.*, 2022) to analyse both common (minor allele frequency, MAF > 0.01) and rare (MAF < 0.01) or very rare (MAF < 0.001) genetic variants using variant- and gene-based strategies. Moreover, we employed an innovative hypothesis-driven strategy focusing exclusively on genetic regions from a newly curated panel of spermatogenesis-related genes to identify monogenic point mutations leading to SPGF.

## Materials and methods

### Patient selection criteria

The GWAS dataset reanalysed in this study included genotype information from a large cohort of European descent, comprising a total of 1274 SPGF patients and 1951 unaffected controls (Cervan-Martin *et al.*, 2022). Of the SPGF patients, 772 were diagnosed with non-obstructive azoospermia (NOA) and 502 with non-obstructive oligozoospermia (NOSO), as described elsewhere (Cervan-Martin *et al.*, 2022). Moreover, the diagnosis and histological subtypes of around half of our study cohort were confirmed after a biopsy performed for testicular sperm extraction (TESE) for use in ART.

Briefly, the criteria for patient selection included semen analysis, physical examination, endocrine profile, genetic screening (Y chromosome microdeletions, mutations in the *CFTR* locus, and karyotype abnormalities), and medical history evaluation in order to exclude known causes of male infertility and consider only SPGF with an idiopathic aetiology. A screening for rare point mutations was not included as it is not commonly used for routine diagnosis (Krausz *et al.*, 2018). Regarding the controls, approximately half were men with normozoospermia, verified through semen analysis, while the other half consisted of population-representative individuals with self-reported biological fatherhood. Written informed consent was obtained from each participant before the study according to the Declaration of Helsinki and approval was received by the Ethics Committee ‘CEIM/CEI Provincial de Granada’ Andalusia Spain at the session held on 26 January 2021 (approval number: 1/21). Additionally, each participating centre received ethical approval and complied with the requirements of their local regulatory authorities and received the corresponding ethical approval and informed consent from all of the participants (Cervan-Martin *et al.*, 2022).

A replication cohort including 297 cases and 480 unaffected controls was recruited following the same criteria.

Therefore, the combination of the discovery and the replication cohorts reached a total of 1571 SPGF patients and 2431 male controls.

### GWAS quality control and imputation

Briefly, the genotyping was performed using the Infinium™ Global Screening Array-24 v3.0 (GSA, Illumina Inc., San Diego, CA, USA). This genotyping array includes 513 547 variants, which ensures a comprehensive coverage of common and rare variants across diverse populations. It also includes 118 826 variants with clinical relevance according to ClinVar, the Pharmacogenomics Knowledgebase (PharmGKB), and the NHGRI-EBI database. Dedicated R packages (Sepulveda, 2020) and the PLINK v.1.9 software (Chang *et al.*, 2015) were used for the necessary quality controls (QCs) on the genotype data and the genetic association analyses. The gcta64 software (Yang *et al.*, 2011) was selected for principal component (PC) analysis, and genotype imputation was carried out in the TOPMed Imputation Server (Das *et al.*, 2016) following the methods implemented in Eagle v.2.4. (Loh *et al.*, 2016) and minimac4 algorithms (Fuchsberger *et al.*, 2015), as described in Cervan-Martin *et al.* (2022).

All polymorphic genetic variants were analysed, but polymorphisms that presented deviations from the Hardy–Weinberg equilibrium in controls (*P *<* *1E−05) were discarded from further analysis. After QC, the Iberian cohort included a total of 627 SPGF patients, 1027 controls, and 19 581 997 polymorphic variants, and the German cohort reached 647 SPGF patients, 924 controls, and 20 484 966 polymorphic variants.

### SPGF-related gene selection

We conducted a thorough search in renowned databases to pinpoint all the potential genes that might play a role in spermatogenesis and spermatogenic failure, as detailed in Supplementary Table S1. In order to identify all the genes involved in the different biological mechanisms underlying these processes, several resources, such as Gene Ontology (GO) (Aleksander *et al.*, 2023), Kyoto Encyclopaedia of Genes and Genomes (KEGG) (Kanehisa *et al.*, 2017), and Reactome Pathway Database (Rothfels *et al.*, 2023) were queried using keywords such as ‘spermatogenesis’, ‘hormonal regulation’, ‘meiotic recombination’, ‘testis’, ‘male reproduction’, etc. Additionally, the loci that had been previously implicated in the development of phenotypes of SPGF, such as NOSO or NOA, and its different histological subtypes, including hypospermatogenesis (HS), germ cell maturation arrest (MA), and Sertoli cell only syndrome (SCO) (Cervan-Martin *et al.*, 2020), were selected from the ‘International Male Infertility Genomic Consortium (IMIGC) database’ (Houston *et al.*, 2021) and the ‘Infertility Disease Database (IDDB)’ (Wu *et al.*, 2021).

**Figure 1.**
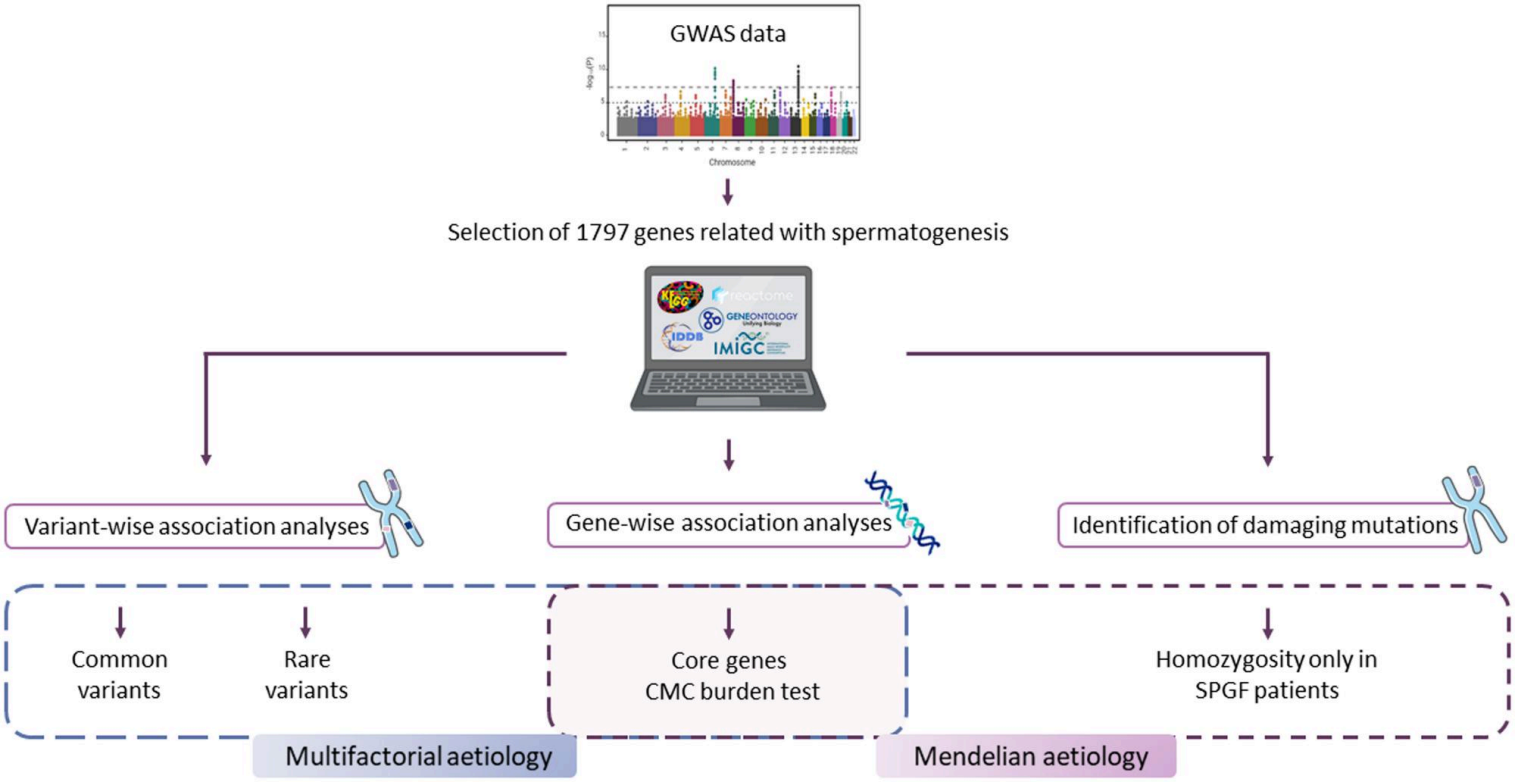
**Overview of the study design.** A panel of 1797 genes related with severe spermatogenic failure (SPGF) were selected and curated, followed by variant-wise, gene-wise, and damaging mutation identification analyses in a large genome-wide association study cohort (GWAS). CMC, combined multivariate and collapsing.

Considering the criteria above, we curated a panel of 1797 unique genes that was used to screen for both common and rare variants potentially implicated in SPGF, considering both multifactorial causes and monogenic factors, as outlined in the study design depicted in Fig. 1.

### Variant-wise association analyses

All polymorphic variants that were located in a ± 10 kb window centred around the selected genes (GRCh38 genome assembly) were included in variant-wise analyses conducted by logistic regressions. The variant-wise approach to the curated gene panel comprised 826 712 polymorphic variants in the Iberian cohort and 764 605 polymorphic variants in the German cohort.

The logistic regression analyses were based on the best-guess genotypes (Rsq > 0.9), assuming additive effects and considering the first 10 PCs and the country of origin as covariates. Then, the different populations were combined into a discovery cohort by conducting an inverse variance meta-analysis considering fixed effects. To measure the heterogeneity of the odds ratios (ORs) across populations, Cochran’s Q and *I*^2^ were estimated. ORs and 95% CI were calculated for all the association tests.

Subsequently, a replication step including an independent case–control population from Spain and Portugal was performed for the top association signals in the discovery cohort. Two SNPs were selected for replication. In this case, the genotyping was performed using the TaqMan™ allelic discrimination technology in a 7900HT Fast Real-Time PCR System (Thermo Fisher Scientific, Pleasanton, CA, USA) and two predesigned TaqMan probes (reference assay IDs: C___1690736_10, C___3260394_10) (Thermo Fisher Scientific). The SDS 2.3 software was used for allele discrimination (Thermo Fisher Scientific). Genetic association was evaluated as described above, and a combined meta-analysis by the inverse variance method was carried out. The significance threshold for these analyses was established at *P *<* *1.12E−05, based on the Bonferroni method to control for multiple testing effects and considering the number of independent variants estimated by the Genetic Type 1 Error Calculator software (Li *et al.*, 2012). We applied a study-wise significance threshold due to our hypothesis-driven approach, which targeted specific genes related to spermatogenic failure and the variants within these regions. This contrasts with a genome-wide and hypothesis-free GWAS analysis, in which more stringent thresholds are commonly used. All the statistical analyses were performed with PLINK v.1.9 (Chang *et al.*, 2015) and R.

Next, a functional characterization of the variants captured by the replicated association signal was performed as previously described (Cervan-Martin *et al.*, 2022; Guzman-Jimenez *et al.*, 2022). Briefly, we analysed the role of the lead variant and its proxies (*R*^2^ > 0.8) as expression or splicing quantitative trait loci according to the v8 GTEx data release (GTEx Consortium, 2020), and their overlap with testis regulatory regions as described in testis-specific ENCODE datasets (Luo *et al.*, 2020). Then, we used online tools such as Haploreg v.4.1 (Ward and Kellis, 2016) and SNPnexus (Oscanoa *et al.*, 2020) to annotate the variants based on several scores for predictive functionality, as detailed in Supplementary Tables S2, S3, and S4. Finally, we performed an enrichment analysis of protein–protein interactions (PPIs) and GO terms with all the bound proteins detected in ChIP-seq experiments and the transcription factors (TFs) with reported binding site (TFBS) sequences affected by the variants using STRINGv11.5 (Szklarczyk *et al.*, 2019).

### Gene-wise association analyses

We implemented a gene-based burden test through the combined multivariate and collapsing (CMC) method (Li and Leal, 2008) to evaluate the cumulative effect of multiple variants located in the same gene. This method allowed us to estimate the combined effect of rare coding variants and low frequency common variants (MAF < 0.05) on SPGF. In this case, the significance threshold was established based on the Bonferroni method and considering the number of independent genes analysed, and it was set at *P *<* *5.56E−04.

### Identification and characterization of highly damaging rare coding variants present in homozygosis

We also decided to follow a strategy focused only on the genetic variants located in the coding sequences (as defined in GENCODE V36, GRCh38/hg38) of the genes in the curated panel. Overlap was calculated using the BEDTools v2.27.1 toolset (Quinlan and Hall, 2010) and PLINK v.1.9 software (Chang *et al.*, 2015). Then, only the variants with a predicted high impact on protein function were selected. The pathogenic consequences were established considering the estimations by the several algorithms, such as (i) sorting intolerant from tolerant algorithm (damaging consequences correspond to score ≤0.05) (Sim *et al.*, 2012), (ii) polymorphism phenotyping (selecting only ‘possibly damaging’ or ‘probably damaging’ variants) (Adzhubei *et al.*, 2010), and (iii) combined annotation-dependent depletion (pathogenic variants show score > 20) (Rentzsch *et al.*, 2019).

Finally, we examined whether those variants were previously described in the CLINVAR, IDDB (Wu *et al.*, 2021), or IMIGC (Houston *et al.*, 2021) databases, and we considered the allele frequency in the general populations included in the TOPMed project (Taliun *et al.*, 2021).

Additional annotations such as Genomic Evolutionary Rate Profiling (GERP++) (Huber *et al.*, 2020), Phylogenetic Analysis with Space/Time Models (PHAST) (Hubisz *et al.*, 2011), structural variations, overlap with regulatory elements, or haploinsufficiency, as reported in DECIPHER v11.23 (Foreman *et al.*, 2023), were retrieved.

## Results

### A 1797 gene panel for SPGF

We carried out a comprehensive search to identify all the possible genes implicated in the molecular pathways related to spermatogenesis by integrating the available information about GO and function or previous associations with male infertility.

**Figure 2.**
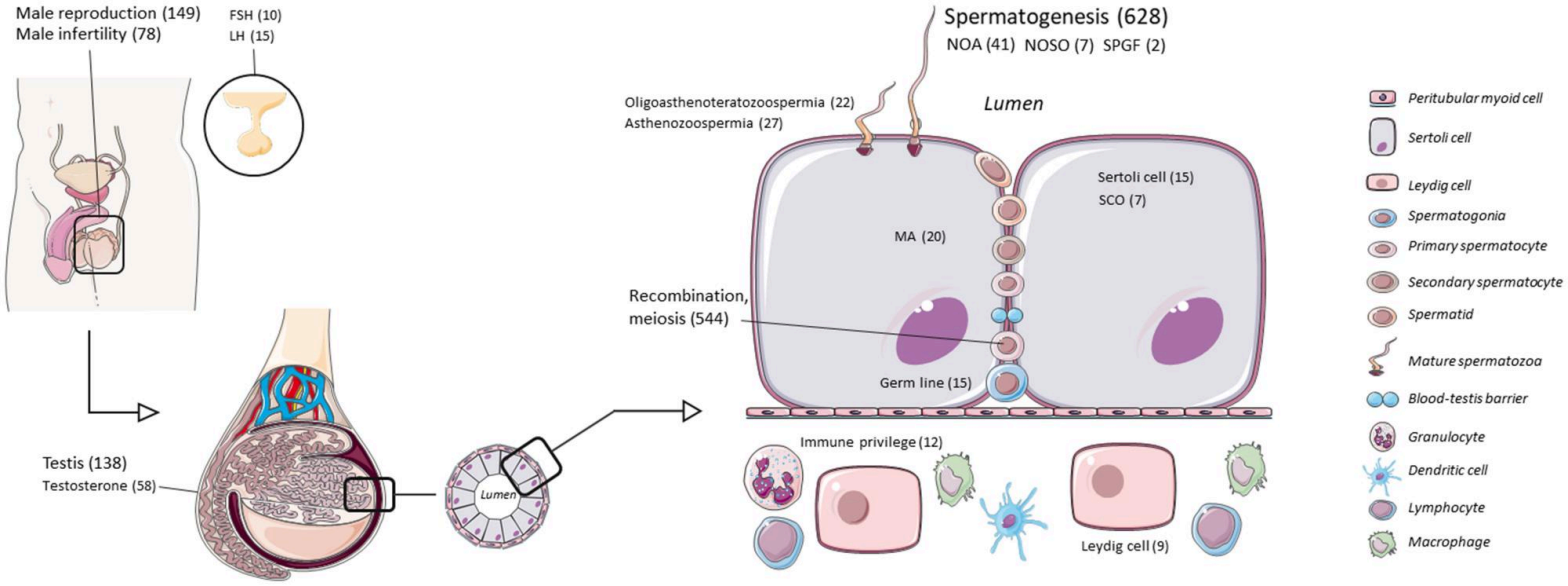
**Graphic representation of the keywords used for the selection of the severe spermatogenic failure (SPGF) gene panel.** Numbers indicate the genes stratified by keyword. MA, maturation arrest; NOA, non-obstructive azoospermia; NOSO, non-obstructive severe oligozoospermia; SCO, Sertoli cell only syndrome.

In detail, we found 628 genes related to the key word ‘spermatogenesis’, 544 genes involved in ‘recombination’ or synonyms, 149 genes associated with ‘male reproduction’, 138 genes linked with ‘testis’, 78 genes with reports of ‘male infertility’, and 12 genes involved in ‘immune privilege’. Additionally, we selected 83 genes related to the hormonal regulation of spermatogenesis, such as testosterone, FSH, or LH. Then 39 genes implicated in the function of the cells of the seminiferous tubules, such as ‘germ cells’, ‘Sertoli cells’, or ‘Leydig cells’ were also included (Fig. 2, Supplementary Tables S1 and S5). Our interrogation of the male infertility dedicated databases, i.e. IMIGC (Houston et al., 2021) and IDDB (Wu *et al.*, 2021), resulted in another 126 genes engaged in SPGF, NOSO, NOA or any of the histological subtypes of NOA (HS, MA, or SCO), or other forms of SPGF affecting sperm motility or morphology, for example: asthenozoospermia, teratozoospermia, or globozoospermia (Fig. 2, Supplementary Tables S1 and S5).

Finally, after eliminating redundancy, we compiled a panel of 1797 genes that represent the current knowledge about the genetic background underlying the formation of functional spermatozoa and that might potentially harbour genetic variants associated with SPGF.

### A low-frequency variant in *SHOC1* is a novel risk locus for SPGF

Aiming to make the most of the high coverage of the rare variants in the studied GWAS cohort after imputation, we decided to analyse the genetic associations with SPGF of both common (MAF > 0.01) and rare variants (MAF < 0.01). Therefore, we tested all the polymorphic variants located in a ± 10 kb window centred in the coding regions of the selected loci. This means, 53 580 rare variants and 313 645 common variants were analysed. The effects in the discovery cohort revealed a significant genetic association of rs7873478, located in the *DMRT1 locus*, with SPGF. The minor allele of this common variant showed a risk effect to develop SPGF (rs7873478*C *P *=* *8.30E−06, OR = 1.27), with consistent ORs and no significant heterogeneity observed between populations (Q = 0.12) (Table 1). Additionally, we found a suggestive association between a haplotype tagged by the rs12347237 variant in the *SHOC1 locus* and SPGF. In this case, the minor allele increased the susceptibility to suffer from SPGF with a strong allele effect (rs12347237*T *P *=* *3.17E−05, OR = 2.94) (Table 1). No significant heterogeneity was observed between the allele effects estimated in both cohorts (Q = 0.97) (Supplementary Table S6).

**Table 1.** Genetic variants in male infertility loci associated with severe spermatogenic failure in the variant-wise analyses.

				Effect Allele Frequency											
				Discovery cohort		Replication cohort		Discovery cohort		Replication cohort		Meta-analysis			
Variant ID	Position (GRCh38)	*Locus*	A1	SPGF	CTRL	SPGF	CTRL	*P*	OR (95% CI)	*P*	OR (95% CI)	*P*	OR (95% CI)	Q	*I* ^2^
rs7873478	9:858429	*DMRT1*	C	0.54	0.48	0.47	0.49	**8.30E−06**	1.27 [1.14–1.40]	3.20E−01	0.90 [0.73–1.11]	3.56E−04	1.18 [1.08–1.30]	0.01	81.11
rs12347237	9:111776287	*SHOC1*	T	0.02	0.01	0.03	0.01	3.17E−05	2.94 [1.77–4.89]	3.70E−02	2.16 [1.05–4.45]	**4.15E−06**	2.66 [1.75–4.03]	0.79	0.00

Significant *P*-values are highlighted in bold.

A1, effect allele; CTRL, controls; GRCh38, Genome Reference Consortium Human Build 38; OR, odds ratio; *P*: *P*-value; SPGF, severe spermatogenic failure.

After a replication step in an independent Iberian cohort, only the signal in *SHOC1 locus* rs12347237*T was associated with SPGF at the nominal level for replication (*P *<* *0.05) (Table 1). Moreover, the combined meta-analysis of all cohorts revealed a significant association in this locus (*P*_combined_ = 4.15E−06, OR = 2.66) (Table 1).

Subsequently, we identified a total of 111 rare variants linked (*R*^2^ > 0.8) with rs12347237, with 6 of them coding variants in the *SHOC1* gene (4 missense and 2 synonymous variants) and 105 located in intronic regions (Supplementary Table S7). Remarkably, several non-coding variants in this haplotype overlapped with known regulatory elements in the adult testis (Supplementary Fig. S1 and Supplementary Table S7). Additionally, we analysed the predicted effects of these variants on TFBSs and found 182 TFs with putatively affected bindings to target sequences. Then, a statistically significant PPI enrichment network including these TFs was observed (*P *=* *1.00E−16). Interestingly, the network was related to GO terms such as ‘developmental process involved in reproduction’ (GO: 0003006; *P *=* *2.54E−09), ‘reproductive structure development’ (GO: 0048608; *P *=* *2.60E−07), ‘reproductive process’ (GO: 0022414; *P *=* *2.86E−07), ‘sex determination’ (GO: 0007530; *P *=* *1.70E−03), ‘positive regulation of male gonad development’ (GO: 2000020; *P *=* *2.90E−03), ‘male sex differentiation’ (GO: 0046661; *P *=* *9.10E−03), ‘spermatogenesis’ (GO: 0007283; *P *=* *3.02E−02), and ‘sexual reproduction’ (GO: 0019953; *P *=* *4.01E−02), amongst others (Supplementary Fig. S2 and Supplementary Table S8).

### Combined effects of rare variants suggest *PCSK4*, *AP3B1*, and *DLK1* as susceptibility genes for SPGF

Testing the association of individual rare variants in non-related individuals can be challenging, despite large sample sizes, due to their low frequency. To increase the statistical power to detect genetic associations, methods like aggregation tests group multiple variants into regions, combining their effects for a more effective analysis and reducing the number of tests (Michailidou, 2018). Therefore, considering the relevant role of rare variants in SPGF, we combined the cumulative effects of rare coding variants (MAF < 0.01) and coding variants with low MAF (MAF ≤ 0.05) by means of CMC burden tests. Using this approach, we revealed the association of three novel loci: Proprotein Convertase Subtilisin/Kexin Type 4 (*PCSK4*, *P *=* *1.04E−04 with seven rare variants identified, all being missense and two of them described as deleterious or probably damaging), Adaptor Related Protein Complex 3 Subunit Beta 1 (*AP3B1*, *P *=* *5.26E−04 with 15 rare variants identified, for being missense and the others being synonymous), and Delta Like Non-Canonical Notch Ligand 1 (*DLK1*, *P *=* *5.48E−04 with five rare variants identified, all of them synonymous) (Table 2 and Supplementary Tables S9, S10, and S11). Additionally, the *SHOC1* gene showed a trend for association under a gene-wise framework (*P *=* *4.88E−03) (Table 2 and Supplementary Table S9). It should be noted that, out of the 17 coding variants that were included in the burden test for *SHOC1*, 6 variants were completely linked to the *SHOC1* signal led by the rs12347237 polymorphism in the variant-wise analysis (Supplementary Tables S7, S10, and S11). Nine of the rare variants in *SHOC1* were missense, and several were predicted as deleterious or damaging by different algorithms (Supplementary Table S11). Moreover, a very rare variant encoding the change of the cysteine residue at position 675 to arginine (rs769778522, Cys > Arg), with a very high linkage disequilibrium (LD) with the rs12347237 variant-wise signal (D’ = 1), was predicted as damaging by several implemented algorithms (Supplementary Table S11).

**Table 2.** Genes associated with severe spermatogenic failure in the gene-wise analyses under the CMC burden test method.

Gene	Range	#Variants	NonRefSite	*P*
*PCSK4*	19:1481427−1490450	7	509	**1.04E−04**
*AP3B1*	5:78000521−78294698	15	389	**5.26E−04**
*DLK1*	14:100726891−100738224	5	71	**5.48E−04**
*SHOC1*	9:111686170−111794937	17	153	4.88E−03

Significant *P*-values are highlighted in bold.

Variants, number of variants analysed; NonRefSite, non-reference site, individuals with other alleles at the evaluated position that are not the reference allele, genotype is not exactly 0 for the reference allele; *P*: *P*-value; range, gene coordinates.

### Novel rare homozygous variants putatively causing SPGF

**Figure 3.**
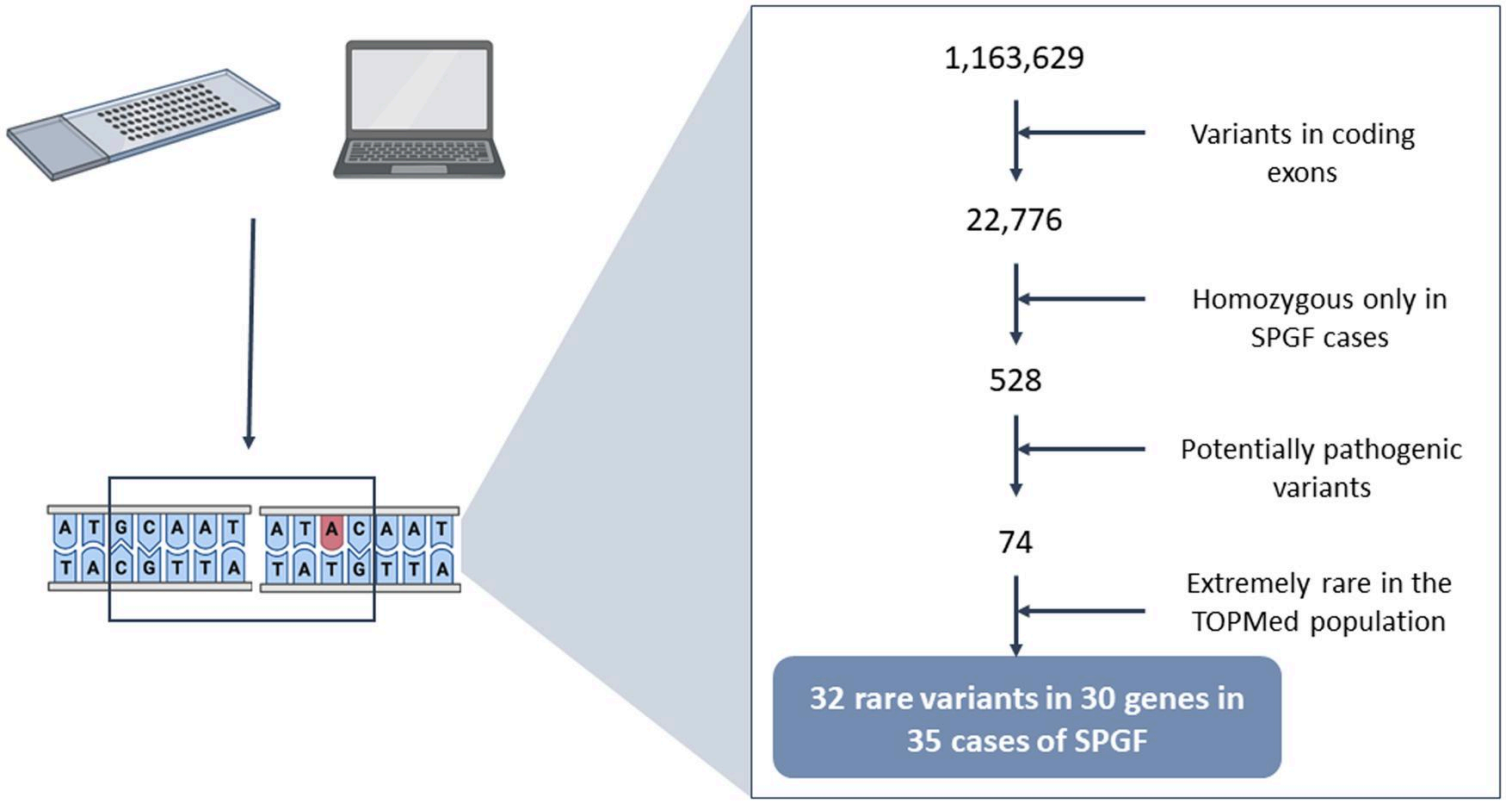
**Rare damaging variant identification using a high-throughput genotyping platform and a genotype imputation strategy.** SPGF, severe spermatogenic failure.

In order to assess the SPGF cases with monogenic aetiology, we identified 32 rare coding variants, 17 on autosomal chromosomes and 15 on the X chromosome, with a high probability of deleteriousness and present in homozygosis only in SPGF patients (Fig. 3 and Supplementary Table S12). These variants were present in 35 SPGF cases (3 Germans and 32 Iberians) and corresponded to 30 unique genes. Interestingly, one of these variants was previously associated with SPGF: rs372254398 (MAF_ALFA_ = 8.00E−05), which is located in the FA Complementation Group A *(FANCA) locus* (a gene with a ‘moderate’ amount of evidence of causing male infertility according to IMIGC). This variant encodes a missense variant that generates an amino acid change (Arg > Gln) at position 880, which was described in NOA patients. Moreover, we found seven rare variants not previously correlated with SPGF in loci that harbour known variants that lead to male infertility (Supplementary Table S13). For example, we reported rs927968522 (MAF_ALFA_ = 2.10E−04), which mapped in the Mastermind Like Domain Containing 1 (*MAMLD1*) gene (a *locus* with ‘strong’ evidence of contributing to male infertility according to IMIGC) and encoded an amino acid change (Pro > Leu) in residue 384 of the protein (Supplementary Table S13). We also found rs79497050 (MAF_ALFA_ = 1.15E−03), which was located in the Sperm Associated Antigen 17 gene (*SPAG17*) and produced a Cys > Phe change in residue 806. Although *SPAG17* showed a ‘limited’ association with asthenozoospermia according to IMIGC, IDDB reported four variants associated with asthenozoospermia or azoospermia, and the knock-out (KO) mouse models for this locus showed reduced sperm numbers with altered motility and morphology despite a normal reproductive system (Xu *et al.*, 2018; Abdelhamed *et al.*, 2020). We identified a variant located in the REC114 Meiotic Recombination Protein (*REC114*) gene, rs371048409 (MAF_ALFA_ = 1.40E−04), which originated from a Pro > Leu change in the 22nd residue. *REC114* has been recently associated with male infertility (Xu *et al.*, 2024) and recurrent pregnancy loss, and KO mice show affected spermatogenesis (Xu *et al.*, 2023). Regarding rs780206976 (MAF_ALFA_ = 8.00E−05), it affected the Telomere Repeat Binding Bouquet Formation Protein 1 (*TERB1*), leading to a Glu > Gly in residue 326. *TERB1* was not included in the IMGC list, but IDDB reports another mutation linked with NOA and male gametogenesis defects in mouse models (Zhang *et al.*, 2022). We found that the rs200844717 variant (MAF_ALFA_ = 1.73E−03) altered the protein encoded by the Dynein Axonemal Heavy Chain 6 (*DNAH6*) gene by a Ser > Tyr change in position 2666. Despite being classified as a ‘limited’ risk factor by IMGC, up to seven different mutations in *DNAH6* have been associated with NOA or multiple morphological abnormalities of the flagella in humans. Additionally, we found two mutations located in male infertility related genes on the X chromosome. A very rare variant chrX:19033639:G:A (MAF = 2.65E−05) caused a Thr > Ile change at residue 90 of the protein encoded by the Adhesion G Protein-Coupled Receptor G2 (*ADGRG2*) locus and rs149433874 (MAF_ALFA_ = 7.00E−05), which resulted in an Ile > Thr change in residue 27 of the protein encoded by the Ubiquitin Specific Peptidase 26 (*USP26*) gene. Remarkably, tens of variants in both genes have been reported as causal variants for NOA previously, and they are considered definitive or moderate risk factors for this phenotype by IMIGC, respectively (Houston *et al.*, 2021).

The remaining variants were located in genes not previously reported as risk factors for human male infertility but clearly linked to the spermatogenic process (Supplementary Table S12). We believe that they are strong candidates as causal variants for the aetiology of SPGF in the corresponding patients, but they should be cautiously considered until further validation in an independent cohort of individuals and/or functional analysis is performed.

Finally, we confirmed that after the removal of the 35 SPGF cases with putative monogenic causes, the association signals detected in the variant-wise analyses showed increased statistical significance (Supplementary Table S6). These findings supported the idea that individuals with monogenic causes of SPGF increase the statistical noise in GWAS analyses focused on the effect of common variation in complex forms of SPGF.

## Discussion

In this study, we aimed to address the contribution of genetic determinants to the development of idiopathic SPGF in a large European cohort by examining, for the first time, the role of two complementary sources of genetic variability: rare and common variants in the human genome. Moreover, we developed a novel framework that was based on a comprehensive search to select SPGF-relevant loci and to implement genetic association analyses exclusively in these regions, both in a variant-wise and a gene-wise fashion. Finally, we demonstrated that it is possible to identify very rare coding variants in homozygosis that can be putatively established as causal variants for SPGF using GWAS datasets instead of next-generation sequencing (NGS) methods (Fig. 1).

Using the variant-wise approach, we tested the possible association of each rare or common variant by comparing the allele and genotype frequencies between the SPGF group and the control population. It is known that the role in SPGF of both types of genetic variation in the same locus can be detected using case–control studies, as was reported, for example, in the Bromodomain-containing protein 7 (*BRD7*) (He *et al.*, 2021), the Testis Expressed 15, Meiosis and Synapsis Associated (*TEX15*) locus (Guzman-Jimenez *et al.*, 2022; Qureshi *et al*., 2023), or the protamine 1 (*PRM1*) gene (Tuttelmann *et al.*, 2010). Our results confirmed that the interrogation of candidate regions in a large SPGF European cohort is also a fruitful strategy to disentangle the genetic component of SPGF, regardless of the MAFs. Our results revealed a trend of association between common variants in the *DMRT1 locus* and a significant association between rare variants in *SHOC1* and SPGF. On the one hand, *DMRT1* is pivotal for male sex determination and the testis development cascade, by preventing the expression of female-specific differentiation pathways (Jimenez *et al.*, 2021; Zarkower and Murphy, 2022). Moreover, rare variants in this locus have been associated with male infertility (Emich *et al.*, 2023). On the other hand, *SHOC1* encodes a key protein for proper meiotic recombination through the formation of crossing-overs and the resolution of meiotic recombination intermediates (Macaisne *et al.*, 2008; Guiraldelli *et al.*, 2018). Furthermore, SHOC1 is necessary for the recruitment of TEX11 and MSH4, which are also involved in meiotic recombination (Guiraldelli *et al.*, 2018). In this sense, *Shoc1*-deficient mice and humans showed profound defects in spermatogenesis leading to SPGF due to MA at the spermatocyte stage (Wang *et al.*, 2022). The modest size of the replication cohort allowed us to confirm the association in *SHOC1*, in spite of its low frequency, due to the remarkable effect size of the risk allele observed for this signal (OR > 2.9). However, the effect of the selected variant on *DMRT1* was considerably weaker (OR < 1.3) and it is likely that the lack of replication might be due to reduced statistical power at this stage. Consequently, future studies are warranted to confirm the possible involvement of common variants in *DMRT1* in idiopathic SPGF.

The gene-wise analyses, through the implementation of CMC burden tests, also yielded very valuable insights. First, we confirmed the relevance of the *SHOC1* locus and identified up to 17 rare and very rare variants that were in high LD with rs12347237, the variant-wise lead polymorphism. In this regard, rare mutations in *SHOC1* were previously associated with MA (Krausz *et al.*, 2020; Yao *et al.*, 2021; Wang *et al.*, 2022). Furthermore, a missense variant in *SHOC1* was established as causal for NOA in a recent report by Wang *et al.* (2022). The reported variant, rs533026166 (NM_173521.5: c.4274G>A), caused an Arg > His amino acid change in position 1425. One of the missense variants analysed in our gene-wise CMC burden test, rs10981009 (c.4273C>T), originates as an Arg > Cys change exactly in the same position, which reinforced the robustness of our approach. Moreover, the combined analyses of rare variants at the gene-wise level allowed us to identify three genes, which were not previously associated with male infertility-related mutations but which play important roles in spermatogenesis. In this regard, *PCSK4* is a highly conserved locus in mammals that encodes a protease expressed only in the testis, placenta, and ovary with an essential role in fertilization (Gyamera-Acheampong *et al.*, 2006; Gyamera-Acheampong and Mbikay, 2009). *AP3B1* is related to spermatogenesis due to its role in endosome and lysosome metabolism (Jing *et al.*, 2019). Finally, *DLK1* encodes a growth regulator involved in infertility and genetic central precocious puberty in males that has been proposed as a link between metabolism and fertility (Rockett *et al.*, 2004; Gomes *et al.*, 2019; Palumbo *et al.*, 2023); interestingly, *DLK1* was found to be overexpressed in testicular tissue from SCO infertile patients (Bonache *et al.*, 2014). The characterization of the functional impact of these loci and the analysis of rare variants in human male infertility will contribute to uncovering novel mechanisms implicated in the deregulation of spermatogenesis that lead to SPGF.

The clinical heterogeneity of SPGF is based on a complex genetic architecture involving several types of rare and common variants, which range from point mutations to large structural variants, and are either inherited or spontaneously generated (*de novo*) (Wagner *et al.*, 2023). Numerous loci have been implicated in the pathogenesis of SPGF by the identification of rare, frequently *de novo*, and potentially deleterious mutations in spermatogenesis-related genes. Nonetheless, the identification of these rare monogenic or oligogenic causal variants relies on expensive NGS techniques and contributes significantly to the risk at an individual level, but constitutes only a minor portion of the overall risk within the affected individuals (Laan *et al.*, 2021). On the contrary, a large proportion of SPGF cases seem to have a complex aetiology, where genetic risk arises from common inherited variants that exert modest individual effects (Visscher *et al.*, 2021). In this context, genotyping using GWAS assays is cheap, but considerable sample sizes are required to identify genetic associations with SPGF. To date, patient cohorts are not routinely screened to identify either monogenic or complex forms of SPGF in a combined and integrative approach. However, we have proven that, based on a previously validated workflow in familial cases of male infertility (Lopez-Rodrigo *et al.*, 2022), it is possible to take advantage of GWAS data not only to analyse common variants involved in complex forms of SPGF but also to detect known rare coding variants in unrelated individuals. Indeed, we currently propose 32 rare potentially pathogenic variants in 35 homozygous patients, one of them in *FANCA* gene, rs372254398, has been reported as a cause for SPGF (Krausz *et al.*, 2019; Tang *et al.*, 2022). We have also identified putatively causal variants in seven genes validated as aetiological factors of male infertility, such as *SPAG17*, *REC114*, *TERB1*, *DNAH6*, *ADGRG2*, *MAMLD1*, and *USP26* (Houston *et al.*, 2021; Wu *et al.*, 2021). Moreover, we found a previously known variant. Although the study of *de novo* variants would still require the implementation of NGS, these findings confirmed the value of GWAS data to identify rare inherited causal variants, which was consistent with the assumption that monogenic causes may explain around 5% of idiopathic male infertility (Cioppi *et al.*, 2021; Cervan-Martin *et al.*, 2022).

Moreover, understanding the biological relevance and mechanisms underlying the role of the identified rare variants will require variant-specific functional experiments and validation through independent replication studies with larger sample sizes (Cano-Gamez and Trynka., 2020). Additionally, our analyses were limited by the availability of data regarding relevant covariates. Given the documented age-related declines in sperm function and the progression from NOSO to NOA over time (Bak *et al.*, 2010; Di Persio *et al.*, 2021), future research should account for age as crucial factor in analysing SPGF.

Finally, it should be noted that 14 genes located in the X chromosome were affected by some of the potentially pathogenic rare causal variants in homozygosis as described in this study. This evidence underscores the substantial contribution of X-linked genetic factors to male reproductive disorders, as men are hemizygous for the X chromosome and mutations in single-copy X chromosome genes lack compensatory mechanisms (Vockel *et al.*, 2021). For example, one of the reported loci, *USP26*, has been proposed as a gene with a moderate to definitive diagnostic value for SPGF in a recent study focused on the role of the X chromosome in idiopathic SPGF susceptibility (Riera-Escamilla *et al.*, 2022).

In summary, we applied a novel and integrative approach to analyse the monogenic and complex aetiology of SPGF in a large European cohort using GWAS genotyping data for variants that were located in a manually curated panel of genes. We were successful in identifying novel risk loci for SPGF at the variant-wise and gene-wise levels and we show that GWAS genotype data was useful to analyse inherited rare variants that putatively cause SPGF in homozygosis. Therefore, we consider that GWAS may continue uncovering the architecture of male infertility, and the contributions of different variants into disease risk. Future research integrating GWAS results with new technologies, such as single-cell genotyping or long-read sequencing, which allow us to consider the impact of individual cell variations in the phenotypic heterogeneity of SPGF, may lead to more personalized diagnostic and therapeutic strategies (Le Bourhis *et al.*, 2000; Cheung *et al.*, 2023). The proposed framework will help with genetic counselling and molecular diagnosis for SPGF patients and support further research to advance the study of the genetic backgrounds of SPGF.

## Supplementary Material

hoae069_Supplementary_Data

## Data Availability

The data generated in this study are either contained in the article file and its supplementary material or available upon reasonable request to the corresponding author.
