# Supplementary figures and images for: A comprehensive study of common and rare genetic variants in spermatogenesis-related loci identifies new risk factors for idiopathic severe spermatogenic failure
Source: Hum Reprod Open. 2024 Nov 13;2024(4):hoae069. doi: 10.1093/hropen/hoae069 (PMC11645127; doi:10.1093/hropen/hoae069)

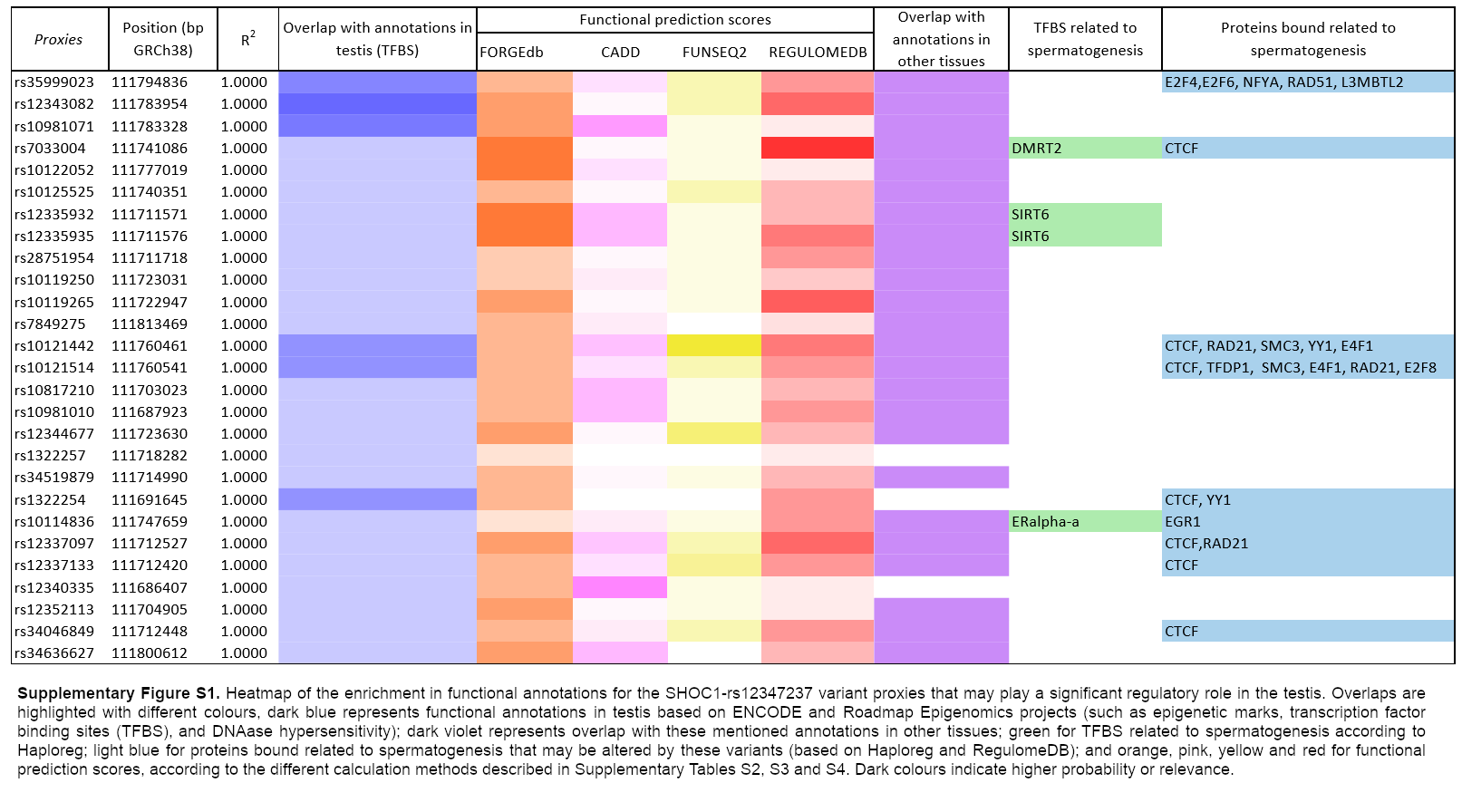

Supplement: hoae069_Supplementary_Data [file hoae069_supplementary_data.zip › SupplementaryFigure1_wLegend.png]

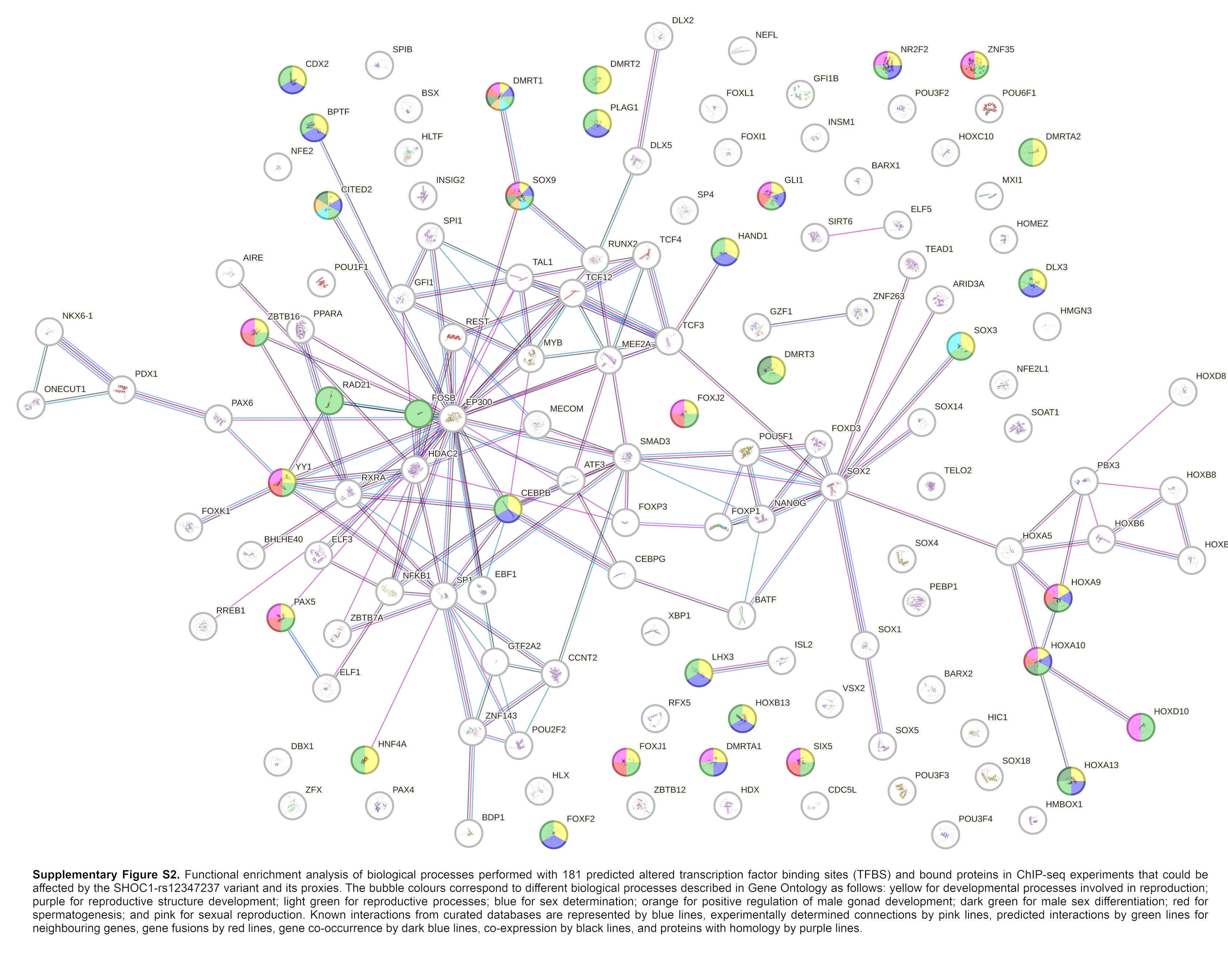

Supplement: hoae069_Supplementary_Data [file hoae069_supplementary_data.zip › SupplementaryFigure2_wLegend.png]
